# CD226 Is Required to Maintain Megakaryocytes/Platelets Homeostasis in the Treatment of Knee Osteoarthritis With Platelet-Rich Plasma in Mice

**DOI:** 10.3389/fphar.2021.732453

**Published:** 2021-08-30

**Authors:** Yongming Liu, Yuan Zhang, Jinxue Zhang, Jingchang Ma, Ka Bian, Yuling Wang, Xuexue Xu, Shuwen Wu, Kun Cheng, Yun Zhang, Yong Ding, Yong Zhou, Ran Zhuang

**Affiliations:** ^1^ Orthopedic Department of Tangdu Hospital, Fourth Military Medical University, Xi’an, China; ^2^ Institute of Medical Research, Northwestern Polytechnical University, Xi’an, China; ^3^ Department of Immunology, Fourth Military Medical University, Xi’an, China; ^4^ Otolaryngology Department of Tangdu Hospital, Fourth Military Medical University, Xi’an, China

**Keywords:** CD226, platelet-rich plasma, platelets, megakaryocytes, osteoarthritis

## Abstract

Platelet-rich plasma (PRP) is a platelet-based application used to treat osteoarthritis (OA) clinically. The co-stimulatory molecule CD226 is expressed in T cells, NK cells, and also platelets. However, exact effects of CD226 on platelets and whether its expression level influences PRP efficacy are largely unknown. Here, CD226^fl/fl^PF4-Cre mice were obtained from mating CD226^ fl/fl^ mice with PF4-Cre mice. Blood samples and washed platelets were collected from the mice eyeballs to undergo routine blood tests and transmission electron microscopy. Differentially expressed proteins were detected by iTRAQ-based proteomics analysis. Animal OA models were established through surgical destabilization of the medial meniscus (DMM) for C57BL/6 wildtype mice, followed by PRP injection to evaluate the effects of platelet CD226 on PRP efficacy. The results showed that deletion of platelet CD226 increased the number of megakaryocytes (MKs) in bone marrow (BM) but reduced MKs in spleen, combined with significantly decreased platelet amounts, α-granule secretion, and reduced immature platelets; indicating that absence of platelet CD226 may disrupt MK/platelet homeostasis and arrested platelet release from MKs. Sequencing analysis showed abnormal ribosomal functions and much downregulated proteins in the absence of platelet CD226. Autophagy-related proteins were also reduced in the CD226-absent MKs/platelets. Moreover, deletion of platelet CD226 diminished the protective effects of PRP on DMM-induced cartilage lesions in mice, and PDGF restored it. Therefore, deficiency of platelet CD226 inhibited platelet maturation, secretion, and normal ribosomal functions, which may lead to depressed PRP efficacy on OA, suggesting that CD226 is required to regulate platelet growth, functions, and its application.

## Introduction

Platelet is well-known to play essential roles in primary thrombosis and hemostasis; In addition, platelet also exerts multiple effects on the regulation of plenty of other pathophysiological processes (Gremmel et al., 2016). As various key cytokines, growth factors, and extracellular matrix modulators (like platelet‐derived growth factor (PDGF), transforming growth factor‐β, insulin‐like growth factor‐I, and epidermal growth factors) were contained in platelets, they have earned much attention in regenerative medicine and applied in tissue regeneration (Scully et al., 2018). Nowadays, platelet-rich plasma (PRP), as one popular platelet application, is widely used in several clinical diseases, especially the skeletal muscle-related damage (Etulain, 2018; Scully et al., 2018).

Osteoarthritis (OA) is a common, debilitating disease, usually accompanied by pain and loss of mobility, and impairs quality of life. This multifactor-induced disease is characterized by destructions of articular cartilage, alterations in subchondral bone, and synovitis (Martel-Pelletier et al., 2016). Currently available drugs for OA treatment are directed predominantly toward the symptomatic relief of pain and inflammation, such as analgesics, nonsteroid and steroid anti-inflammatory drugs, glucosamine, chondroitin sulphate, and hyaluronic acid (HA). However, they do little to reduce joint cartilage degeneration (Spaková et al., 2012). It was showed that PRP could ameliorate arthritis via suppression of inflammation and oxidative stress (Araya et al., 2020; Ragab et al., 2021). Intraarticular PRP injections provide a simple, low-cost, and minimally invasive method to enhance tissue regeneration by providing autologous blood growth factors with normal concentrations (Filardo et al., 2011; Smelter and Hochberg, 2013). To date, intraarticular PRP therapies have been used clinically for over 10 years in arthritis, as a safe and effective method for injured knee and cartilage repair treatment through aiding tissue regeneration and reducing inflammation (Kon et al., 2009).

However, due to the lack of suitable optimization and standardization techniques for PRP preparation, many different platelet-rich products with controversial therapeutic effects have been developed (Leitner et al., 2006). It was reported that thrombin activation of PRP inhibited chondrogenesis and osteogenesis. Nonactivated PRP resulted in increased formation of bone and cartilage *in vitro* and *in vivo* (Han et al., 2009). Thus, as no definitive recommendations have been made until now, the status of PRP (e.g., pH and activation status) should be monitored to enable a comparison across studies and therapy modalities.

Platelet functionally expressed immune checkpoint molecules, such as the ligand of murine glucocorticoid-induced tumor necrosis factor receptor (GITRL) and platelet/endothelial cell adhesion molecule 31 (CD31), which regulate tumorigenesis, cardiovascular disease, and inflammatory disease (Li et al., 2017; Zhou et al., 2021). CD226 is an immunoglobulin-like glycoprotein, and mainly identified as a co-stimulatory immune receptor on T and NK cells via its ligands CD112/CD155 (Catros et al., 2014; Sanchez-Correa et al., 2019). CD226 expression pattern in the platelet and its involvement in platelet activation was demonstrated in 1989 (Scott et al., 1989). CD226 also functioned in megakaryopoiesis and platelet formation (Gieger et al., 2011). However, the exact role CD226 plays in MKs/platelets and platelet-mediated diseases is still largely unknown.

The aim of current study was to assess the function of CD226 in megakaryocytes (MKs)/platelets, to determine whether this adhesion molecule affects the efficacy of PRP and its application on the amelioration of joint damage induced by surgical destabilization of the medial meniscus (DMM)-caused knee OA in a mice model.

## Materials and Methods

### Mice

All procedures and protocols were approved by the Scientific Research Ethics Committee of the Fourth Military Medical University. All experiments were performed in accordance with the principles and guidelines of the Care and Use of Laboratory Animals. Wildtype C57BL/6 mice (WT) were obtained from the experimental animal center of Fourth Military Medical University. CD226^fl/fl^ mice with C57BL/6 background were constructed by the Cyagen company (Suzhou, China). Platelet factor 4 (PF4)-Cre mice were obtained from the model animal research center of Nanjing University. CD226^fl/fl^ mice were breed with PF4-Cre mice to acquire CD226^fl/fl^PF4-Cre mice with specific knockout of CD226 in megakaryocytes (MKs)/platelets. The CD226^fl/f^PF4-Cre mice were genotyped via classical polymerase chain reaction (PCR) for PF4-Cre (forward primer sequence: CCC​ATA​CAG​CAC​ACC​TTT​TG; reverse primer sequence: TGCACAGTCAGCAGGTT) in DNAs extracted from tail, and CD226 deletion was checked via flow cytometric analysis in CD41^+^ platelets isolated from mice (antibodies used were CD41a-FITC and CD226-APC (eBioscience, CA, United States). The mice were housed under specific pathogen-free conditions in standard, with individually ventilated cages, and were fed with standard laboratory chow and water. All mice that were used in this study were 8–12 weeks old (weight 23–26 g).

### Preparation of Platelet-Rich Plasma and Washed Platelets

CD226 ^fl/fl^ and CD226^fl/f^PF4-Cre mice were anesthetized with 2.5% isoflurane. When mice were in a coma and muscles were relaxed, blood samples were collected from the eyeballs into EP tubes with EDTA anticoagulant powder. PRP was separated at the upper layer from the blood samples by centrifugation at room temperature (RT) for 10 min at 160 g/min. For the preparation of washed platelets, PRP was centrifuged at 2,000 g/min for 10 min at RT, and the platelet pellet was resuspended (Zhang et al., 2020).

### Histological Analysis of Megakaryocytes in Mice Bone Marrow and Spleen Tissues

Mice were sacrificed after anesthesia. Femurs were taken out, and muscles and fascia were removed, followed by fixation with 4% paraformaldehyde, decalcification with 10% EDTA, dehydration with ethanol in different concentration, and paraffin embedding. Embedded tissues were then cut into slices with 4 μm thickness. Paraffin embedding of spleen tissues need no decalcification. The slices were stained with Hematoxylin and Eosin dye (Servicebio, Wuhan, China) following manufacturer’s instruction. After staining, EVOS M5000 microscope was used to observe and capture images. MK amounts and area were analyzed by QuPath software.

### Measurement of Platelet Number and Maturation by Flow Cytometry

Platelet number was evaluated by flow cytometry through staining with a FITC-conjugated anti-mouse CD41 antibody (#11–0,411-85, eBioscience). The calculation formula of platelet amounts was as: platelet number = the whole blood cells × the percentage of platelet analyzed by flow cytometry. For platelet maturation detection, Sulfo-NHS-LC-Biotin solution (Sigma-Aldrich, Missouri, Unites States) was used to label all blood cells through intravenously injection. From day 1 to day 5, blood (15 μl) was collected from tail vein after the injection, and biotin-positive cells were detected by phycoerythrin (PE)-conjugated streptavidin (#MA1-20010, eBioscience). Platelets stained with APC-CD41 antibodies (#17–0,411-82, eBioscience) were distinguished from other blood cells. Subsequently, blood cells were incubated with 1 μg/ml thiazole orange (TO; Sigma-Aldrich) dye for 15 min at RT to stain immature reticulated platelets and fixed with formaldehyde, followed by three-color flow cytometric analysis to determine the immature/mature percentage of platelets from day 1 to day 5 as previously reported (Zhang et al., 2020).

### Ultrastructural Observation of Platelets

Washed platelets were fixed in 0.1 M cacodylate buffer containing 2.5% glutaraldehyde for 90 min, and 1% acrylamide for 90 min. After dehydration, fixed platelets were embedded in epon, and cut into 70–80 nm slices by ultramicrotome (Leica, Vienna, Austria). Slices were transferred onto the copper grid. Uranyl acetate and lead citrate were used to stain sections, which were observed and analyzed under a CM120 transmission electron microscope.

### Routine Blood Tests and Enzyme-Linked Immunosorbent Assay

A total of 50 µl blood samples were collected into tubes containing EDTA. Routine blood tests were performed using a CLVC DF-3000Vet automated hematology analyzer (Bejing, China), and following parameters were detected: white blood cells (WBCs), red blood cells (RBCs), hemoglobin (HGB), mean platelet volume (MPV), platelets (PLT), plateletcrit (PCT), platelet distribution width (PDW), platelet-large cell count (P-LCC) and platelet-large cell ratio (P-LCR). The serum concentrations of PDGF‐AB and platelet factor 4 (PF4) were analyzed using their corresponding ELISA kits (#EK0486 for PDGF-AB, #EK0727 for PF4; Boster, Wuhan, China) according to the manufacturer’s instructions.

### Isobaric Tags for Relative and Absolute Quantification (iTRAQ)

Collected platelet protein samples were also used to perform iTRAQ-based proteomics analysis. In brief, platelets were dissolved in lysis buffer and labeled with iTRAQ-labeling reagents. High-Performance Liquid Chromatography (HPLC) Pump system (LC-20AB, Shimadzu, Kyoto, Japan) coupled with a high pH RP column was used to separate peptides. The peptides separated from nano HPLC were subjected to tandem mass spectrometry Q Exactive HF X (Thermo Fisher Scientific, San Jose, CA, United States) for data-dependent acquisition detection through nano-electrospray ionization. Labeled peptides with isobaric tags were then quantitatively analyzed by an automated software IQuant (Wen et al., 2014). Results with a Q value <0.05 and fold change >1.2 were set as the significant threshold for differential expression, and number cutoff values of three for quantifiable peptides were accepted for in-protein abundance.

### Primary Culturing of Megakaryocytes From Mice Bone Marrow Cells and Dami Cell Culture

Dulbecco’s modified eagle medium (DMEM, Gibco, United States) was used to flush the BM cavity to receive BM cells, that were then filtered through a 70 μm cell strainer to remove spicules and clumps. Murine MKs were generated by culturing BM cells in DMEM containing 10% fetal bovine serum (FBS, Gibco), 1% Penicillin/streptomycin (Gibco), and 50 ng/ml thrombopoietin (TPO, Novoprotein, Shanghai, China) for 3 days. MKs were then separated from marrow cells using a two-step BSA gradient (Schulze, 2016), and resuspended with TPO medium to further incubate for 2 days. Additionally, Dami (human megakaryocytic) cell line was obtained from the American Type Culture Collection (Manassas, USA). Cells were maintained in RPMI-1640 medium supplemented with 10% FBS at 37°C with 5% CO_2_ and saturated humidity. For CD226 gene silencing, lentivirus carrying CD226 shRNA (shCD226, 5′- GCA​CTG​TGT​GAA​GAG​ACA​TTG-3′) or its scramble control (NC, 5′- ACT​ATA​TAT​CGT​CCT​TAA​GCT-3′) was synthesized by Orbialgene Co. Ltd. (Xi’an, China) and was used to infect the cells as previously reported (Zhang et al., 2019).

### Western Blot

The total proteins were isolated from tissues, cells or washed platelets using a radioimmunoprecipitation assay lysis buffer containing 1 mM protease/phosphatase inhibitor cocktail (Cell Signaling Technology, CST, MA, United States). Protein concentration was determined by BCA protein assay kit (Beyotime, Shanghai, China). Afterward, 30 μg protein was resolved by SDS-PAGE and transferred onto PVDF membranes (Millipore, United States). After blocking, membranes were incubated with primary antibodies: anti-CD226 (1:500, #50232-RP02, Sino Biological, Beijing, China), anti-Beclin1 (1:500, #sc-48341, Santa Cruz Biotechnology, Heidelberg, Germany), anti-LC3 (1:500, #sc-398822, Santa Cruz Biotechnology), anti-S6 ribosomal (1:1,000, #2217, CST), anti-PDGF-A (1:500, #sc-9974, Santa Cruz Biotechnology), anti-PDGF-B (1:500, #sc-365805, Santa Cruz Biotechnology), and anti-β-actin (1:3,000, #T0022, Affinity, United States) overnight at 4 °C, and subsequently incubated with corresponding secondary antibodies for 1 hour at RT. The blots were visualized with enhanced chemiluminescence (ECL) reagents. Quantification of the blot intensities was conducted using Image Lab 6.1 software (BioRad, Hercules, CA, United States).

### Immunofluorescence

Femur tissues were fixed in 4% paraformaldehyde and decalcification using 10% EDTA. After dehydration, tissues were embedded in a 1:1 mixture of sucrose and optimal cutting temperature compound (OCT, Servicebio) for 2 h, then equilibrated in OCT for 5 h, followed by freezing quickly. After permeabilization with 0.5% TritonX-100 and blocking with goat serum (Boster), cryostat sections (5 µm) were incubated with primary antibodies including anti-von Willebrand factor (vWF, 1:100, #ab11713, Abcam, Cambridge, United Kingdom), anti-Beclin1 (1:100, #sc-48341, Santa Cruz Biotechnology) at 4°C overnight. On the other hand, TPO-induced MKs were incubated with anti-S6 ribosomal (1:200, #2217, CST) at 4°C for 10 h. Corresponding secondary antibodies were used to incubate sections at RT for 1 h in dark. Nuclear counterstaining was performed using DAPI (Boster). After mounting, the sections were captured by a EVOS M5000 immunofluorescence microscopy and analyzing by Qupath software.

### Establishment of Osteoarthritis Animal Model and Platelet-Rich Plasma Treatment

Mice OA models were surgically induced by DMM in healthy male C57/BL6 mice, which involved transection of the medial meniscotibial ligament. Under general anesthesia with pentobarbital sodium (0.5 mg/10 g body weight, i. p.), the hair of the right hind limbs was shaved for the preparation of aseptic surgery. Using this model, the lesions progressed to severe OA at 8 weeks post-surgery, as previously reported (Hoshi et al., 2017). To observe the effects of CD226 in platelet on PRP treatment for OA, C57BL/6 mice were randomly divided into six groups: Sham-operated (*n* = 4), DMM + saline (*n* = 8), DMM + HA (*n* = 8), DMM + PRP from CD226^ fl/fl^ mice (*n* = 8), DMM + PRP from CD226^fl/fl^PF4-Cre mice (*n* = 8), and DMM + PRP from CD226^fl/fl^PF4-Cre mice + PDGF-AA (*n* = 8). Surgery was performed on the right knees of each mouse, as previously described (Glasson et al., 2007). The same procedure was performed in the sham-operated group without transection of the medial meniscotibial ligament. All intraarticular injections were applied under 2.5% isoflurane anesthesia, with a 1-ml insulin syringe (BD Company). Each mouse received four consecutive injections of 10 μL of saline, PRP, or HA. The first injection was given 4 weeks after DMM surgery, and consecutive injections were repeated every week until 8 weeks after DMM surgery. The concentration of PDGF-AA used was 10 μg/ml (Lohmander et al., 2014). During the entire experiment, the animals were allowed to move, drink, and eat *ad libitum.*

### Histological Analysis for Joint Damage

Histological assessment of the articular cartilage was performed at the eighth week post DMM surgery. In the sagittal plane, paraffin-embedded sections of 5-μm thickness were made at approximately 50-μm intervals throughout the whole knee joint. The slides were stained with hematoxylin and eosin (HE) and Safranin O-fast green dye (Serviobio) according to the manufacturer instruction. The articular cartilage lesions were then blindly assessed following Osteoarthritis Research Society International (OARSI) histological scoring system (Glasson et al., 2010): the higher the score, the severer the lesions.

### Statistical Analysis

Data are presented as mean ± SD. Data were analyzed using independent-sample *t*-tests for comparisons between two groups. The score-related data were analyzed using the Kruskal–Wallis test followed by Dunn’s multiple comparison test. Statistical significance was defined as *p* < 0.05. Statistical analyses were performed with GraphPad Prism 6 (GraphPad Software, La Jolla, CA, USA).

## Results

### Deletion of Platelet CD226 Disturbs the Homeostasis of Megakaryocytes/Platelets

**FIGURE 1:**
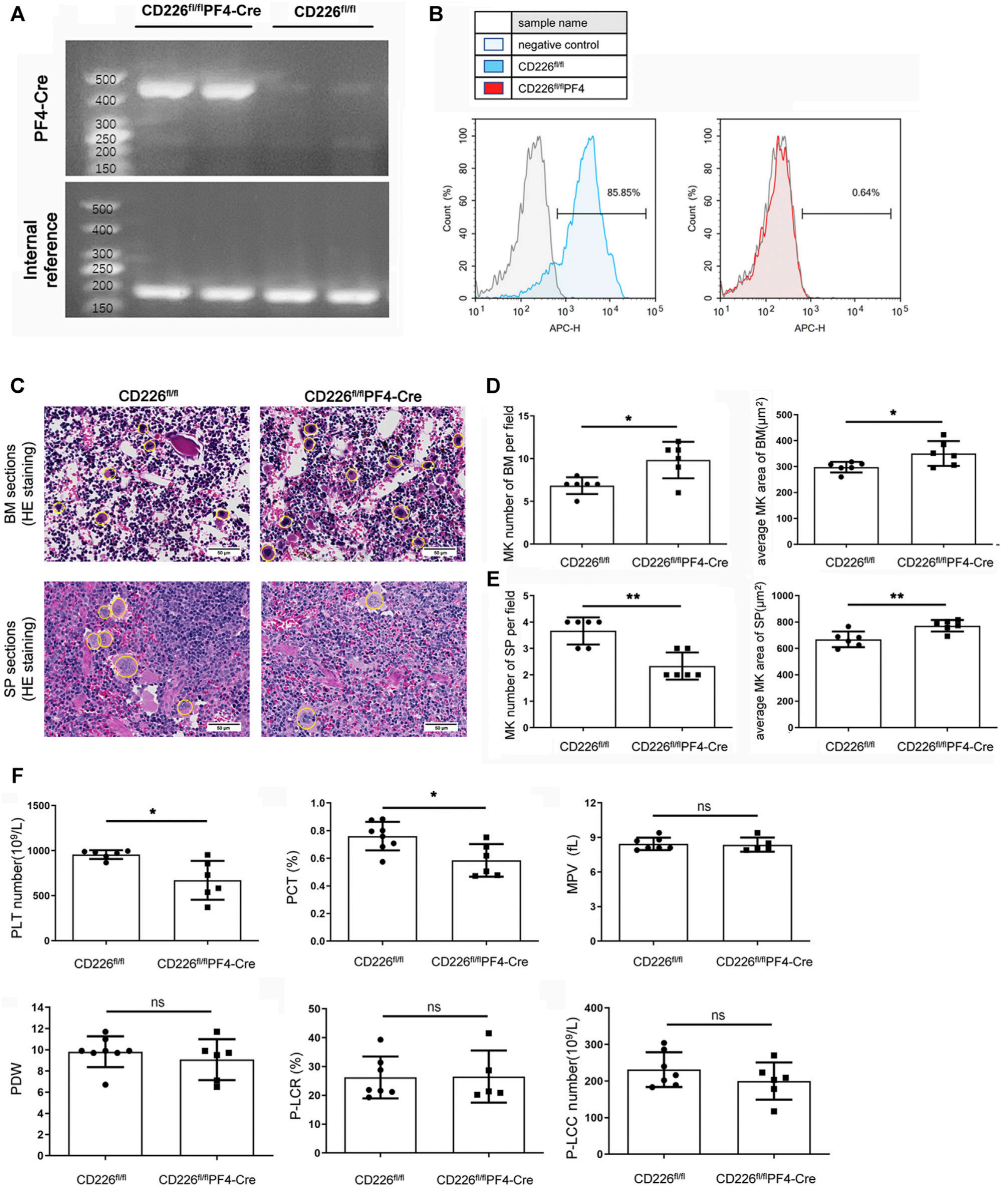
Deletion of platelet CD226 disturbed the homeostasis of MKs/platelets. **(A)** Identification of CD226^ fl/fl^ and CD226^fl/fl^PF4-Cre mice through PCR of DNAs extracted from the tail. **(B)** Flow cytometry images showing CD226 expression in CD226^ fl/fl^ and CD226^fl/fl^PF4-Cre mice. **(C)** HE staining of BM and spleen sections. The MKs are circled; scale bar = 50 µm. **(D, E)** MK number and average MK area in BM and spleen sections. **(F)** Routine blood test results of platelets (PLT) number, plateletcrit (PCT), mean platelet volume (MPV), platelet distribution width (PDW), platelet-large cell ratio (P-LCR), and platelet-large cell count (P-LCC). Statistical significance between groups was analyzed using independent-sample *t*-tests with two-tailed *p* value. *p < 0.05, **p < 0.01, ns = no statistical significance.

MK/platelet-specific CD226 knockout mice (CD226^fl/fl^PF4-Cre mice) were constructed and genotyped via classical PCR of DNA extracted from tail (Figure 1A). The deletion of CD226 was evaluated in CD41^+^ platelets by flow cytometry (Figure 1B). Further flow cytometric analysis indicated that the deletion of CD226 was in a tissue specific manner, CD226 expression levels in B cells, T cells, NK cells, CD11b^+^ myeloid cells and CD11c^+^ conventional DC was comparable in splenocytes of mice (Suppl. Figure 1). We firstly detected the MK numbers in BM and spleen tissues from CD226^ fl/fl^ and CD226^fl/fl^PF4-Cre mice (Figure 1C). It was shown that MK/platelet-specific absence of CD226 significantly increased MK counts in the BM of the femur; but decreased MK counts in the spleen compared to CD226^ fl/fl^ mice (Figures 1D,E). The average MK areas in both BM and spleen were significantly increased in CD226^fl/fl^PF4-Cre group, compared to that in littermates (Figures 1D,E). These findings suggested that platelet CD226 deficiency may induce a hyperactivity status of MKs in the central immunological organ to compensate for the dysfunction of MKs/platelets in peripheral immune system. In addition, routine blood test of blood samples from CD226^ fl/fl^ and CD226^fl/fl^PF4-Cre mice showed that CD226 deletion in platelets significantly reduced platelet (PLT) counts and plateletcrit (PCT) compared to those in littermates (Figure 1F). However, other platelet-related parameters and blood components exhibited no significant difference between the two groups (Figure 1F, Supplementary Figure S2A).

### Absence of CD226 in Platelets Hinders Platelet Maturation and Reduces α-granule Secretion

**FIGURE 2:**
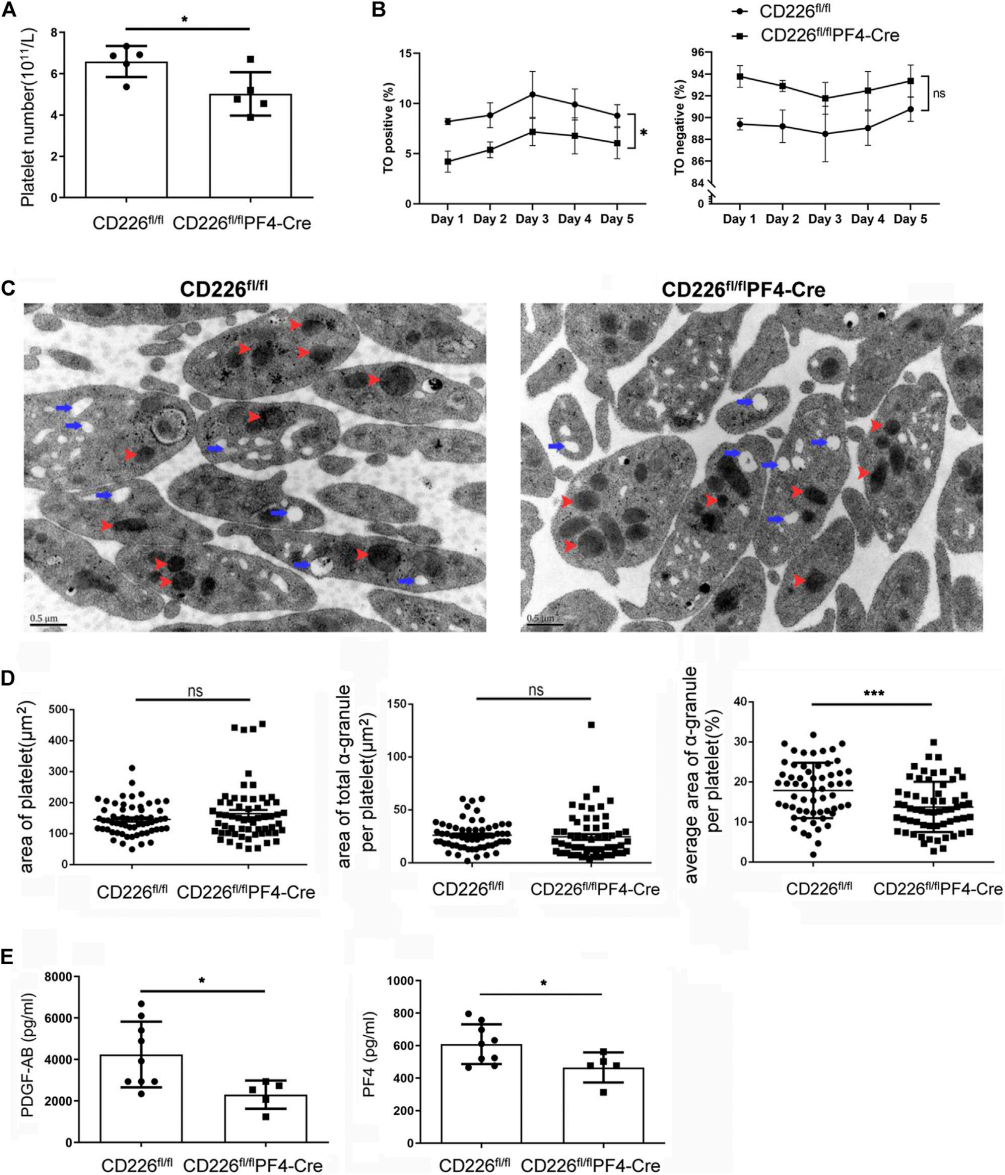
Absence of CD226 in platelets hindered platelet maturation and reduces α-granule secretion. **(A)** Platelet number was evaluated by flow cytometry. **(B)** Percentage of TO positive and negative platelets was measured to show the maturation of platelets. Two-ANOVA was adopted for statistical analysis over time. **(C)** Morphological changes of platelets. Red arrow: α‐granule; blue arrow: dense-granule. **(D)** Area of platelet, area of total α‐granule per platelet, and average area of total α‐granule per platelet were calculated. **(E)** Concentrations of PDGF-AB and PF4 in serum. Statistical significance was analyzed by independent-sample *t*-tests with two-tailed *p* value. *p < 0.05, ***p < 0.001, ns = no statistical significance.

Flow cytometry analysis was carried out to confirm the declined platelet amounts after CD226 knockout in platelets (Figure 2A). To figure out why, the platelet maturation in CD226^ fl/fl^ and CD226^fl/fl^PF4-Cre mice was determined through TO staining in a time course. Newly born or immature platelets containing RNAs were labelled with TO, while aged or mature platelets were TO negative. It was shown that deletion of platelet CD226 significantly decreased the immature platelet proportion (TO+); although TO- platelets showed increased ratio, there was no statistical significance between the two groups (Figure 2B). This observation suggested that platelet CD226 deletion hindered the maturation of platelets. Moreover, ultrastructural morphologic changes in the resting platelets of CD226^fl/fl^PF4-Cre and littermates were observed using transmission electron microscopy. Platelets contain three major types of secretory organelles: lysosomes, dense (δ)-granules, and α‐granules. We found that the overall structure of platelets was intact, and the area of platelets in CD226^fl/fl^PF4-Cre was similar to that in the littermates (Figures 2C,D). Although the area of total α-granule per platelet had no change, the average area of α-granule per platelet was significantly reduced in CD226^fl/fl^PF4-Cre mice (Figure 2D). The total and average area of δ-granules in platelets from the two groups exhibited no difference (Suppl. Figure 2B). Platelet α-granules may secrete adhesive proteins and growth factors involved in hemostasis, as well as glycoproteins involved in inflammation, wound healing, and tissue regeneration (Maynard et al., 2007). Herein, the levels of secreted PDGF-AB and PF4, which were abundant in platelets, were evaluated through ELISA. It was shown that both PDGF-AB and PF4 were significantly reduced in the CD226^fl/fl^PF4-Cre mice compared with those in CD226^ fl/fl^ group (Figure 2E), suggesting that CD226 deficiency in platelets may reduce the α-granule secretion in platelets.

### Platelet-specific CD226 Absence Leads to Abnormal Ribosomal Function and Structure

**FIGURE 3:**
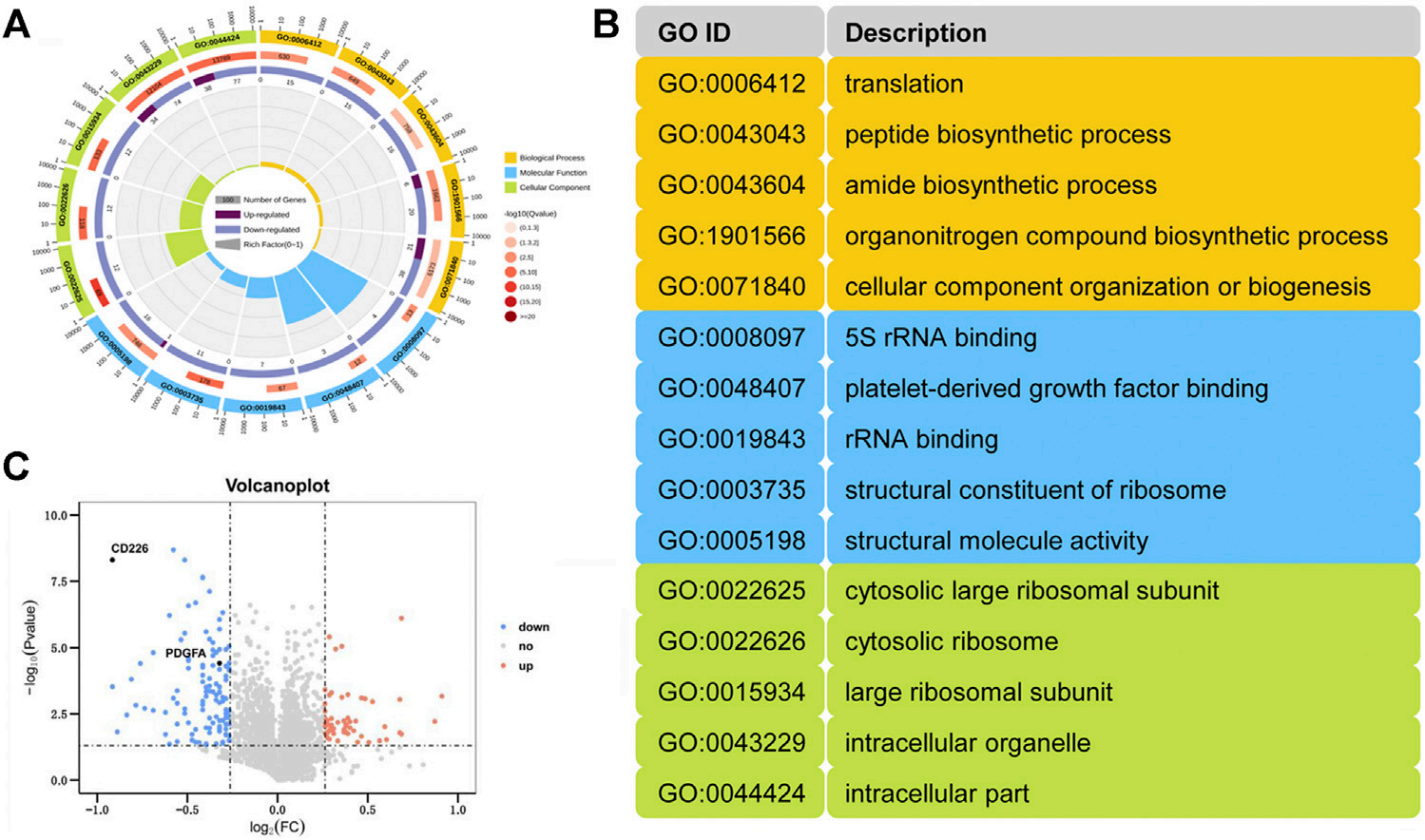
Platelet-specific CD226 absence led to abnormal ribosomal function and structure. **(A)** A GO analysis of the DEPs. **(B)** Annotation of the gene ID. **(C)** Volcano plot of differentially expressing proteins from the CD226^fl/fl^PF4-Cre platelet versus littermates (*n* = 3 per group).

Subsequently, iTRAQ-based quantitative proteomics analysis was performed to explore possible mechanism behind CD226 knockout in platelets. A total of 4,143 proteins were detected. Compared to the CD226^fl/fl^ group, the expression levels of 170 proteins (51 upregulated and 119 downregulated) in the CD226^fl/fl^PF4-Cre group were significantly different. The circle plot showed the top five differentially expressed genes in each category of gene ontology (GO) enrichment (Figure 3A). Top five pathways in the category of biological processes (yellow), molecular functions (blue), and cellular components (green) were illustrated in Figure 3B, which indicated that platelet CD226 deficiency strongly affected the ribosomal functions and structure. A volcano plot of differentially expressed proteins in the CD226^fl/fl^PF4-Cre mice platelets indicated much more down-regulated proteins than up-regulated ones, in which CD226 and PDGF-A were also significantly decreased in the CD226^fl/fl^PF4-Cre mice platelets (*n* = 3 per group) (Figure 3C).

### CD226 Deficiency in Platelets Decreases the Ribosome and Autophagy Related Protein Expression in Megakaryocyte/Platelets and Human Dami Cells

**FIGURE 4:**
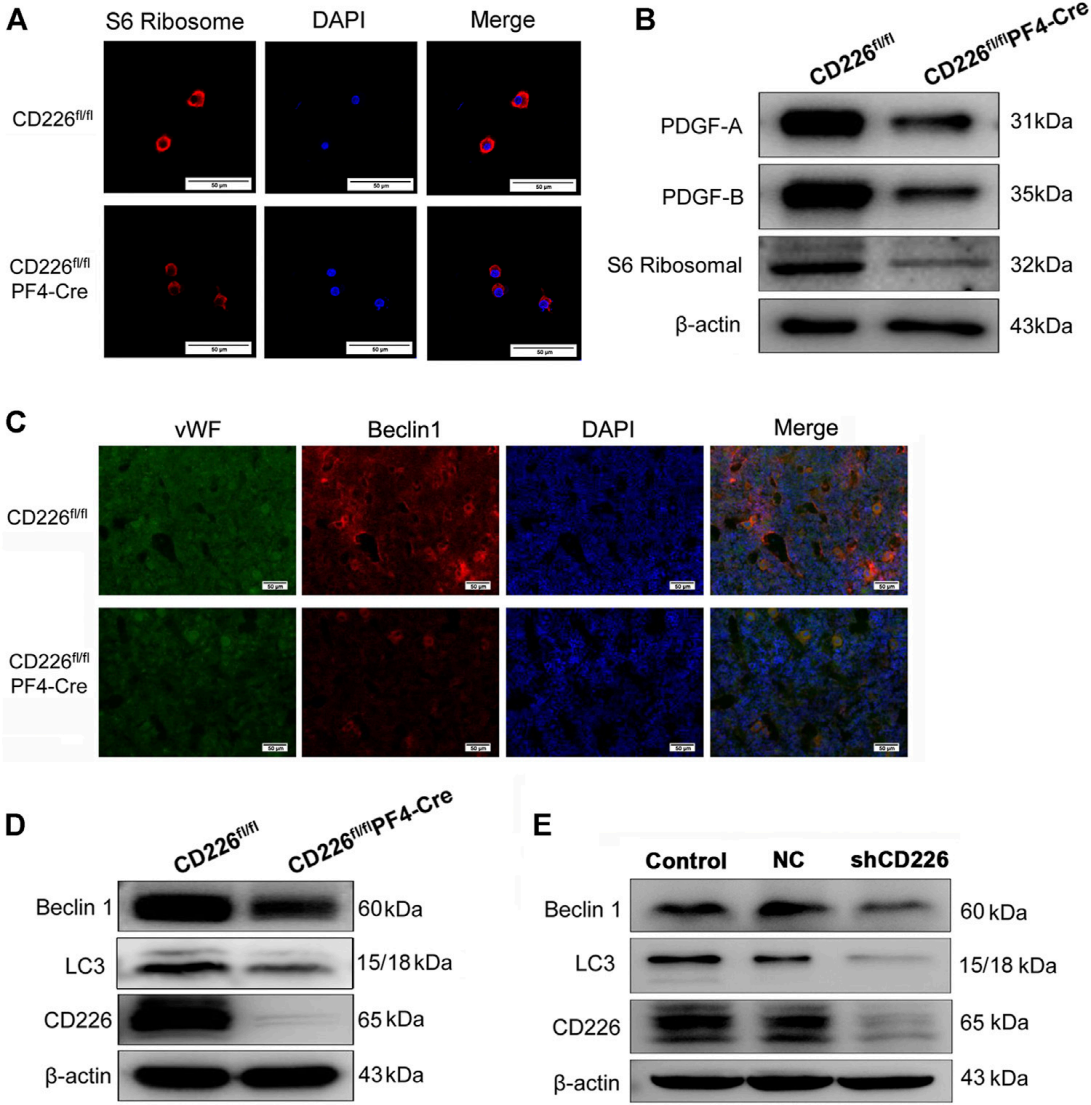
CD226 deficiency in platelets decreased the ribosome and autophagy related protein expression in MK/platelets and human Dami cells. **(A)** S6 ribosomal protein-expressing ribosome in isolated MKs from BM. Scale bar = 50 µm. **(B)** Lysates of freshly isolated mice platelets were subjected to western blot with anti-S6 ribosomal, PDGF-A, and PDGF-B antibodies. **(C)** Double immunofluorescence staining for vWF and Beclin1 in the BM slides from CD226^fl/fl^PF4-Cre and CD226^ fl/fl^ mice. Scale bar = 50 µm. **(D)** Lysates of freshly isolated mice platelets were subjected to western blot with anti-LC3, Beclin1, and CD226 antibodies. **(E)** Dami cells infected with lentivirus shCD226 were subjected to western blot with anti-LC3, Beclin 1, and CD226 antibodies.

The immunofluorescence staining of S6 ribosome in the isolated MKs of BM from CD226^fl/fl^PF4-Cre and CD226^ fl/fl^ mice showed that CD226 deficiency obviously decreased the S6 ribosomal protein levels in MKs (Figure 4A). Further western blot verified the reduced PDGFs and S6 ribosomal protein levels in the CD226^fl/fl^PF4-Cre mice platelets (Figure 4B). In addition, as autophagy is closely related to the MK size and platelet activation (Yang et al., 2016), here we also evaluated autophagy status after CD226 knockout in platelets. BM slides from CD226^fl/fl^PF4-Cre and CD226^ fl/fl^ mice were double-stained with vWF/Beclin1, which showing that morphologically, Beclin1 was at a lower level in MK/platelets of CD226^fl/fl^PF4-Cre mice (Figure 4C). Western blot also showed that Beclin1 and LC3 expression in the platelet (containing leukocytes less than <0.01% through platelet purity assessment) from CD226^fl/fl^PF4-Cre mice was less abundant (Figure 4D). Moreover, human megakaryocytic cell line Dami infected with shCD226 lentivirus was adopted to detect the autophagy level *in vitro*. Like the results in mice platelets, CD226 knockdown significantly decreased the autophagy related protein Beclin1 and LC3 in the megakaryocytic cells (Figure 4E).

### Deletion of Platelet CD226 Diminishes the Protective Effects of Platelet-Rich Plasma on Articular Cartilage of Destabilization of the Medial Meniscus Mice, and Platelet‐Derived Growth Factor Restores It

**FIGURE 5:**
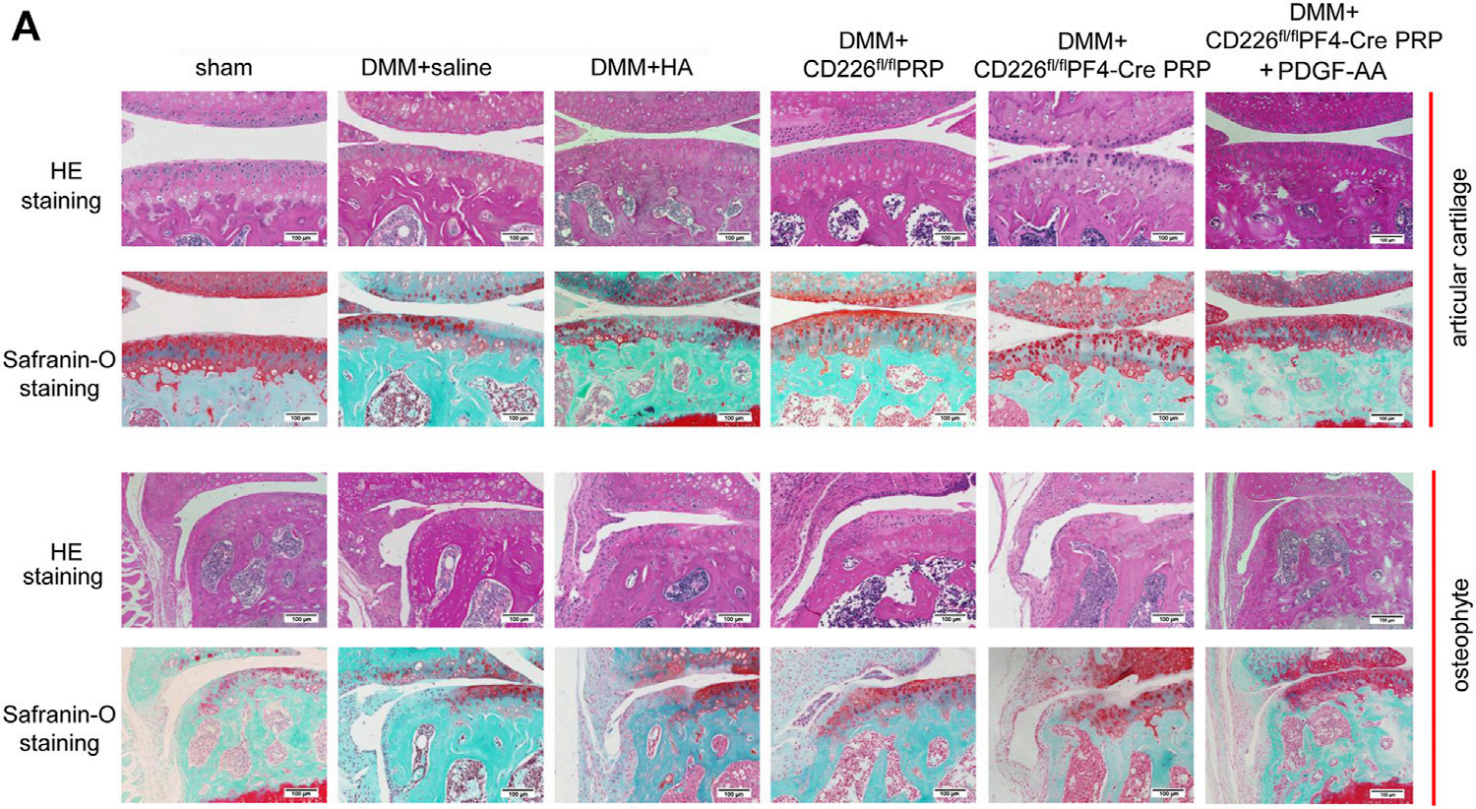
Deletion of platelet CD226 diminished the protective effects of PRP on articular cartilage of DMM mice, and PDGF restored it. **(A)** HE and safranin O-fast green staining of sagittal sections of the medial compartment of the tibia. Scale bar = 100 µm.

**FIGURE 6:**
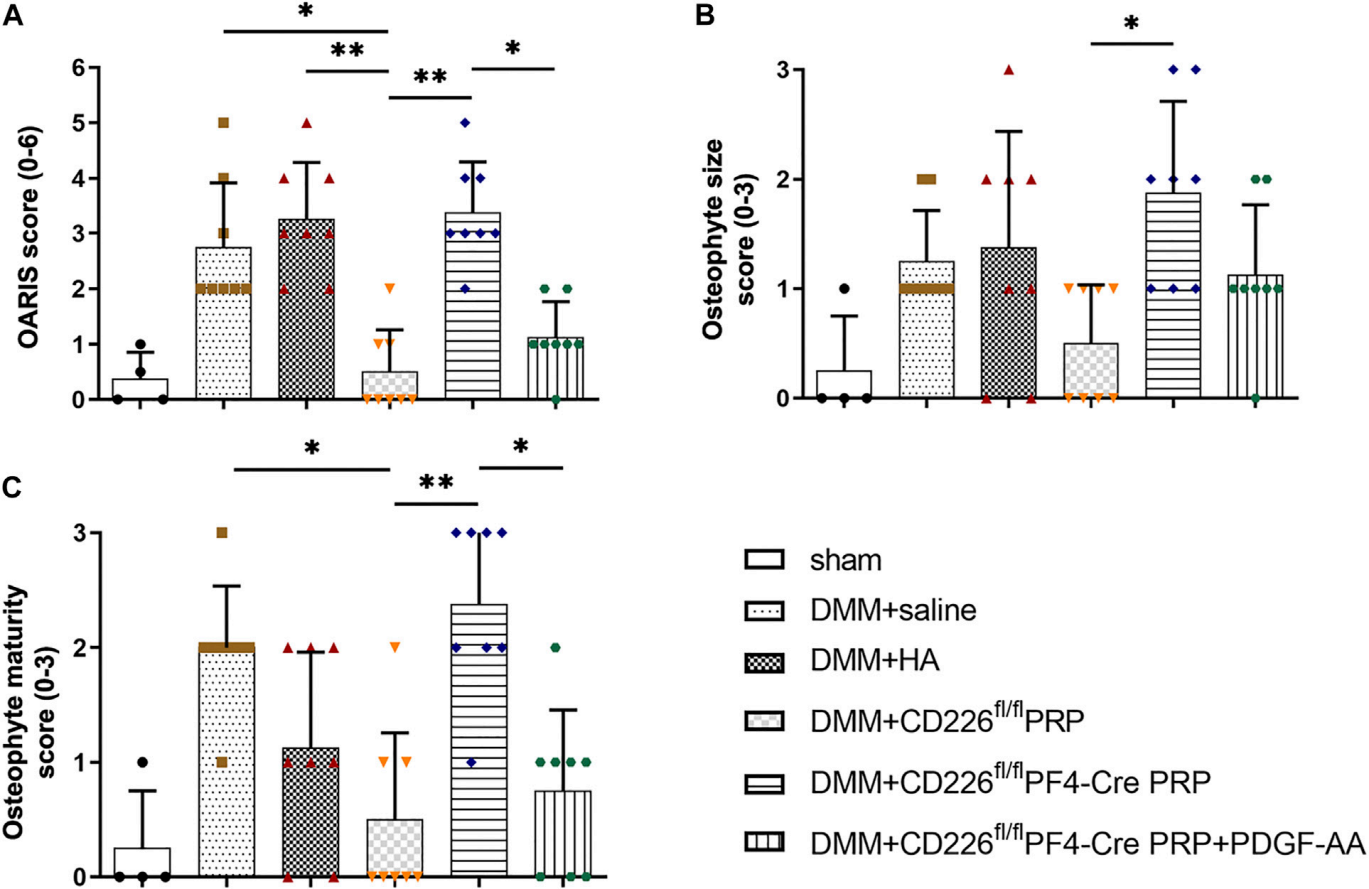
Histological scoring of PRP-treated DMM mice. **(A)** OARIS-modified Mankin score of articular cartilage. **(B)** Osteophyte size scores and **(C)** Osteophyte maturity scores. The score-related data were analyzed by Kruskal–Wallis test accompanied with Dunn’s multiple comparison test. *p < 0.05, **p < 0.01, ns = no statistical significance. Supplementary Figure S1. Flow cytometry analysis for the expression of CD226 in splenocyte from CD226^ fl/fl^ and CD226^fl/fl^PF4-Cre mice. **(A)** Gating strategy for flow cytometric analysis of splenocyte subsets. **(B)** Representative image of CD226 expression levels in B cells, T cells, NK cells, CD11b^+^ myeloid cells and CD11c^+^ DC. Supplementary Figure S2. Other parameter detected after platelet CD226 deficiency in mice. **(A)** Routine blood test of the hemoglobin (HGB), red blood cell (RBC) number, and white blood cell (WBC) number. **(B)** Area of total dense-granule per platelet and average area of dense-granule per platelet in two groups. Statistical significance between groups was analyzed using independent-sample *t*-tests with two-tailed *p* value. ns = no statistical significance.

In order to make a direct connection between CD226 knockout in platelets and its clinical application, we treated DMM-induced mice OA animal models with PRP from CD226^fl/fl^PF4-Cre and CD226^ fl/fl^ mice to detect the possible roles of platelet CD226 in the OA treatment. The results of HE and Safranin O-fast green staining indicated that DMM administration caused severer cartilage defects, massive osteophyte formation, and a significant loss of proteoglycans, with OARIS scores increasing dramatically (Figures 5,6A). Further treatment of normal PRP remarkedly relieved the cartilage defects compared with the saline and HA treatment, which could be aggravated again when administrating the PRP with the absence of platelet CD226 (Figures 5, 6A). Other pathological results of the osteophyte size (0–3 scale) and maturity (0–3 scale) exhibited similar tendency with the OARIS scores (Figures 6B,C). Nevertheless, intraarticularly injection of PDGF-AA into the DMM + CD226^fl/fl^PF4 PRP mice showed restored effects of platelet CD226 deficiency-induced impairment of PRP protective impact on DMM mice. These results demonstrated that platelet CD226 is required in the clinical applications of PRP treatment for OA.

## Discussion

PRP contains fundamental protein growth factors that were proven to be actively secreted by platelets to initiate mesenchymal tissue healing. These growth factors stimulate cell proliferation, migration, differentiation, and matrix synthesis and can affect chondrocyte metabolism and chondrogenesis, as well as improve cartilage healing *in vivo*. It was believed that PRP can augment or stimulate healing with the same biologic healing process that normally occurs in the human body after injury. Biodegradable materials such as gelatin hydrogel microspheres containing PRP indicated that the sustained release of growth factors contained in PRP has preventive effects against OA progression due to the stimulation of cartilage matrix metabolism (Saito et al., 2009). The pooled improvements among different injection therapies were investigated recently. Chou et al. discovered that 3-injection PRP therapy in the treatment of mild-to-moderate knee OA had the best efficacy for pain relief and function improvement compared to one- and 2-injection therapies (Chou and Shih, 2021). To date, the preparation and usage of PPR were not unified, the dosage and frequency of use were also different, and the follow-up time of clinical research was short, so a consensus on the curative effect of PRP had not been formed. In addition, some uncertainties about the efficacy of this method also need to be confirmed, such as the quality of platelets, platelet residence time, systemic reaction, operator injection technology, as well as the functional state of platelet transfusion. These uncertainties lead to debates on PRP treatment for OA (Ornetti et al., 2016). Hence, to explore new methods for prolonging platelet activity and specific targets for monitoring PRP functions in the OA treatment are urgent clinical problems to solve.

CD226 was expressed in CD34^+^ cells derived from fetal liver, fetal bone marrow, and adult bone marrow, as well as in megakaryocyte lines induced by thrombopoietin (Ma et al., 2005). CD226 monoclonal antibody can stimulate platelet activation and aggregation, which causes sustained platelet aggregation independent of granule secretion; its mechanism is similar to collagen-induced aggregation (Zuzel et al., 1991). In our recent research, CD226 was highly expressed in platelets and involved in the development and activation of platelets (Zhang et al., 2019). Further study indicated that the deletion of CD226 resulted in mild thrombocytosis and increased MK/platelet number. Platelet hyperactivity was partially due to the decreased platelet-derived nitroxide level in CD226 global knockout mice (Zhang et al., 2020). These findings indicate that CD226 is a potential regulator for intravascular platelet activation to be regarded as antithrombotic agents.

We used the MK/platelet-specific CD226 knockout mice to elucidate the effects of CD226 on platelets. We found that CD226 deficiency in platelets led to increased MKs in the central immune organ but decreased MKs in the peripheral spleen. Moreover, both routine blood test and flow cytometry indicated that platelet number was reduced in the absence of platelet CD226. These results suggested that CD226 is essential to modulate the MK/platelet homeostasis. Absence of CD226 may induce a hyperactivity status of MKs in the central immunological organ which can further compensate for the declined amounts of MKs/platelets in the peripheral immune system. Deletion of platelet CD226 also showed significantly reduced TO positive platelet over time, which means newly born or immature platelets from MKs were reduced. These findings also verified the previously detected declined platelet numbers. Hence, the balance of MKs/platelets was broken by deletion of platelet CD226, leading to arrested platelet release from MKs.

Within the platelets, α-granules exhibited reduced average areas with decreased secretion levels of PDGF-AB and PF4 growth factors in the absence of platelet CD226. Besides, both KEGG and GO enrichment indicated that deficiency of platelet CD226 led to extensive abnormal ribosomal structures and functions. S6 ribosomal protein and Beclin1 expression was also decreased in MKs and platelets in CD226^fl/fl^PF4-Cre mice versus CD226^ fl/fl^ mice. The proteome of cells is synthesized by ribosome, and dysregulation of ribosome biogenesis directly affects cell development (Teng et al., 2013; de la Cruz et al., 2015). The volcano plot of differentially expressed proteins in platelets also showed more down-regulations than up-regulations, which indirectly confirmed reduced ribosomal function for protein synthesis caused by deletion of platelet CD226. In our previous study, we found that MKs and platelets obtained from global CD226 KO mice had a more dilated tubular membrane system versus WT mice, which might lead to ribosomopathies (Zhang et al., 2020). Hence, the adhesion molecule CD226 has a close relation with ribosomal functions, which should be taken into consideration in the further experiments. In addition, Feng et al. confirmed the existence of autophagy in anucleated platelets, that was closely associated with platelet adhesion, aggregation, and thrombosis (Feng et al., 2014). Yang et al. demonstrated that deletion of mTORC1 suppressed the age-associated elevation of MK size and differentiation, as well as platelet function in mice, indicating that elevated autophagy reduced MK size and repressed platelet activation (Yang et al., 2016). In current study, we observed decreased autophagy in both platelets and MKs with CD226 deficiency in platelets. Combined with the reduced protein synthesis, CD226 in platelets also declined autophagy-related proteins in mice. On the other hand, human MK cells with CD226 knockdown indicated similar effects of CD226 molecule on the cell autophagy, suggesting that CD226 can exert impacts on MK/platelet autophagy not only in rodent but also in human. Therefore, absence of CD226 in platelets significantly reduced α-granule secretion like PDGFs and PF4 growth factors; ribosomal protein and functions in MK/platelet were also obviously decreased, combined with declined autophagy in the platelet CD226 knockout mice.

Although clinical outcomes and efficiency of PRP-administrated OA were variable, PRP is still widely used in the knee OA treatment (Zahir et al., 2021). It is known that the underlying mechanism of PRP to treat knee OA is mainly to stimulate immune cell activation and trigger cell proliferation and inflammation through release or secretion of various growth factors (Szwedowski et al., 2021). Sundman et al. have demonstrated that when synovium and cartilage harvested from OA patients were co-cultured with PRP or HA, the media concentration of TNF-α was reduced compared with the controls (Sundman et al., 2014). Osterman et al. showed that treatment with PRP significantly decreased expression of tissue inhibitor of metalloproteinases-1 and thrombospondin motifs-5 in cartilage, increased aggrecan expression in cartilage, suggesting that PRP injection may be an effective alternative anti-inflammatory agent in OA treatment (Osterman et al., 2015). Therefore, we preliminarily considered that as an intra-articular treatment method, PRP mainly exerts a local effect on synoviocytes and cartilage, nevertheless, this viewpoint needs further research support. Here, our study showed that PRP injection strongly alleviated the cartilage damage induced by DMM in a mice model, however, injection of PRP from platelet-specific CD226 knockout mice abandoned this protective effects. Combined with previous findings, we proposed that deletion of platelet CD226 suppressed platelet maturation and secretion, leading to reduced valuable growth factors and ribosomal functions, thereby diminishing the positive effects of PRP for OA treatment.

In conclusion, absence of platelet CD226 disturbed the homeostasis of MKs/platelets, which hindered platelet maturation and growth factor secretion. Abnormal ribosomal functions with lower protein synthesis and reduced cell autophagy in platelets were also induced by platelet CD226 deletion. These features resulted in depressed effects on PRP efficacy for DMM-induced OA models, leading to serious cartilage lesions in mice. Therefore, CD226 is a crucial molecule in platelets to regulate their normal growth and functions, and it is required for PRP injection to treat OA disease.

## Data Availability

The raw data supporting the conclusions of this article will be made available by the authors, without undue reservation.
