# Supplementary material for: CD226 Is Required to Maintain Megakaryocytes/Platelets Homeostasis in the Treatment of Knee Osteoarthritis With Platelet-Rich Plasma in Mice
Source: Front Pharmacol. 2021 Aug 30;12:732453. doi: 10.3389/fphar.2021.732453 (PMC8436152; doi:10.3389/fphar.2021.732453)
Supplement: Supplementary file 1 [file DataSheet1.PDF]

Supplementary Material

1 Supplementary Figures

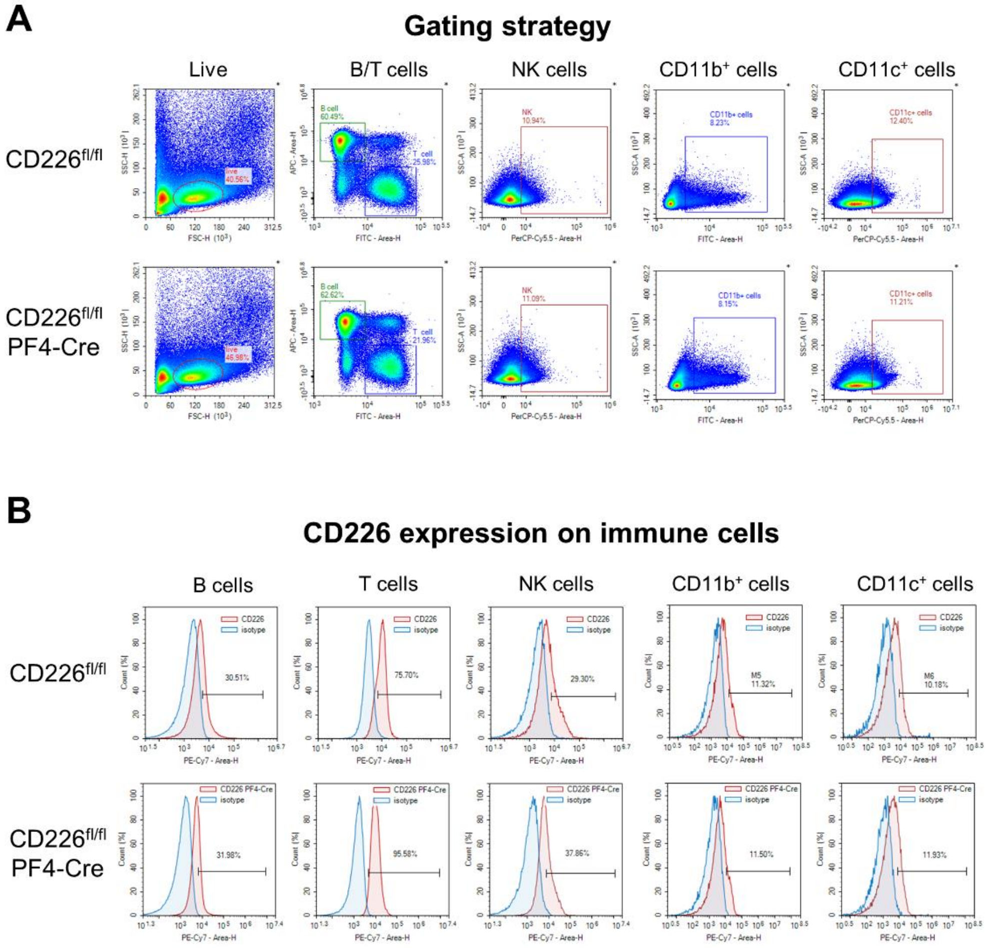

Supplementary Figure 1. Flow cytometry analysis for the expression of CD226 in splenocyte from CD226$^{fl/fl}$ and CD226$^{fl/fl}$PF4-Cre mice. **(A)** Gating strategy for flow cytometric analysis of splenocyte subsets. **(B)** Representative image of CD226 expression levels in B cells, T cells, NK cells, CD11b^{+} myeloid cells and CD11c^{+} DC.

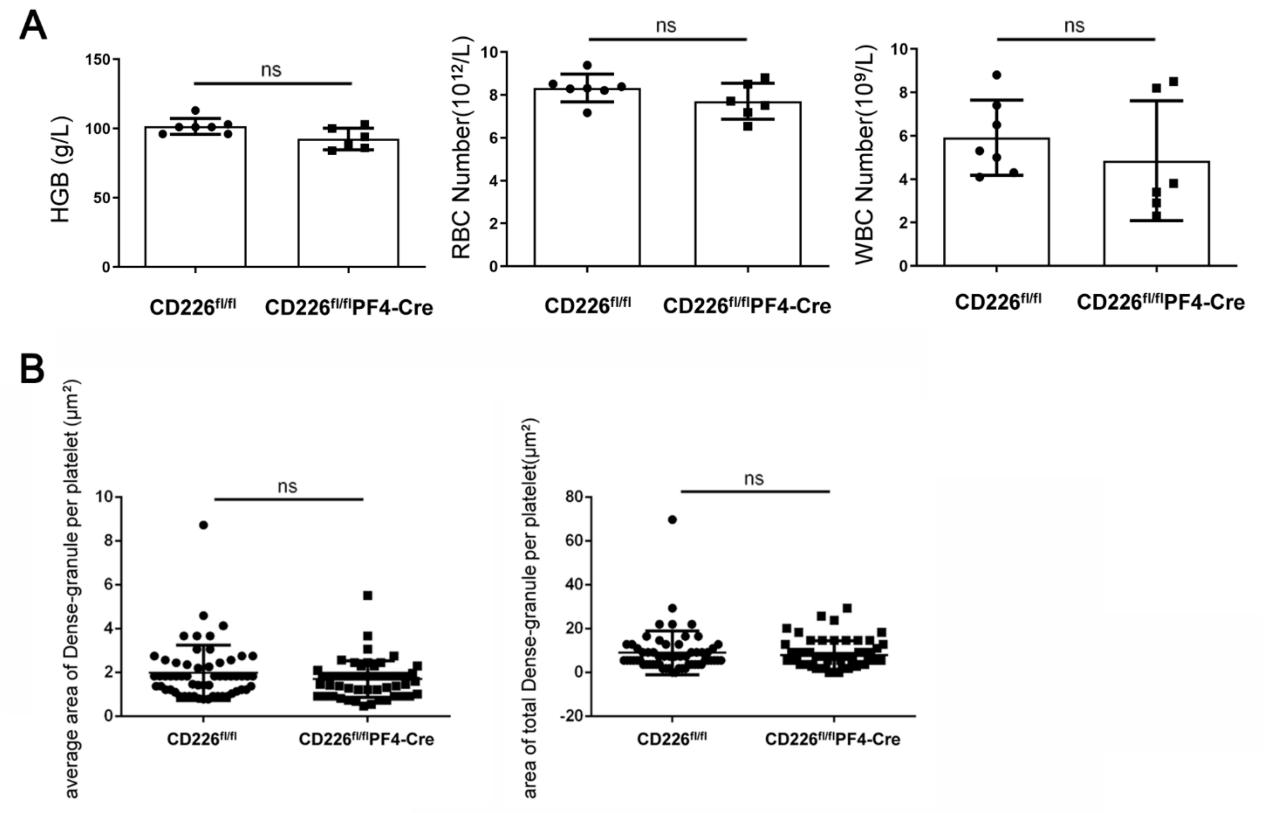

Supplementary Figure 2. Other parameter detected after platelet CD226 deficiency in mice. **(A)** Routine blood test of the hemoglobin (HGB), red blood cell (RBC) number, and white blood cell (WBC) number. **(B)** Area of total dense-granule per platelet and average area of dense-granule per platelet in two groups. Statistical significance between groups was analyzed using independent-sample *t*-tests with two-tailed *P* value. ns = no statistical significance.
